# Ice Cream: new virtual reality tool for the assessment of executive functions in children and adolescents: a normative study

**DOI:** 10.3389/fpsyg.2023.1196964

**Published:** 2023-09-22

**Authors:** Manuel Antonio Fernandez, Fidel Rebon-Ortiz, Miguel Saura-Carrasco, Gema Climent, Unai Diaz-Orueta

**Affiliations:** ^1^Instituto Andaluz de Neurología Pediátrica, Sevilla, Spain; ^2^Giunti-Nesplora SL., Donostia-San Sebastian, Spain; ^3^Department of Psychology, Maynooth University, Maynooth, Ireland

**Keywords:** neuropsychological assessment, virtual reality, executive functions, ecological validity, normative data

## Abstract

This study focuses on the obtention of normative data for participants between 8 and 16 years old who were administered the Ice Cream test, a virtual reality tool designed to evaluate executive functions. The normative sample comprised *n* = 821 participants (49% female), with an age range of 8 to 16 years old, recruited across nine different testing sites in Spain. Experienced evaluators in psychological assessment, recruited and trained by the developer of the test, administered the test to the recruited sample. An empirical analysis of Ice Cream identified three factors, namely planning, learning and flexibility. Descriptive normative groups by age and gender were initially provided. A homoscedasticity analysis by gender showed no statistically significant differences between male and female participants. Cluster analysis by age suggested the creation of different age groups, respectively, 8 to 11 and 12 to 16 in Planning and Flexibility, and 8 to 9 and 10 to 16 in Learning, and subsequently, descriptive data for the established age groups per factor are shown. A confirmatory factor analysis showed the suitability of the 3 factors established as measured of three differentiated executive functions. Complementary data on the validity and reliability, and internal consistency of the scales are provided. Obtained normative data are relevant for evaluating executive functions in children and adolescents in a more ecological way. Further studies are needed to determine sensitivity and specificity of Ice Cream VR test to measure executive functions in different clinical populations.

## 1. Introduction

Executive functions are the set of processes that regulate self-control capacity of our conscious and unconscious systems when it comes to establishing response patterns, organization, planning, time management and, in general, achievement of goals and objectives (Best and Miller, 2010; Bausela-Herreras, 2014; Josman and Meyer, 2018; Ruiz-Gutiérrez et al., 2020). In general, terms like executive functioning or control refer to essential mental abilities to deploy an efficient, creative and socially accepted behavior. In addition, executive functions include a series of cognitive processes, such as anticipation, goal selection, planning, behavior selection, self-regulation, self-control, and feedback (Díaz-Orueta et al., 2014). As accurately described by Diamond (2013, p. 135), they are a series of “top-down mental processes needed when you have to concentrate and pay attention, when going on automatic or relying on instinct or intuition would be ill-advised, insufficient, or impossible” and she refers to the three core EFs as inhibition, working memory, and cognitive flexibility, with all potential name variations associated to these. Previously, Miyake et al. (2000b) acknowledged the relevance of recognizing both the unity and diversity of executive functions, and with their study, they shed some light on the uniqueness of three target executive functions (namely, “shifting”, “updating” and “inhibition”) while recognizing their moderate correlation with one another.

Executive functions (EF) are essential for an adequate neurological development through different life stages (Best and Miller, 2010). Given their role as regulators of multiple processes, both at a cognitive and an emotional level, their correct development is crucial for achieving milestones associated with age in the areas of learning, behavior and emotional management (Bausela-Herreras, 2014). A suboptimal performance of executive functions can condition maturational changes, global performance and the course of a normative or neurotypical development. More specifically, a dysfunction in executive functions may be linked with symptoms associated with developmental disorders such as attention deficit disorder with/without hyperactivity (ADHD) or autism spectrum disorders, among others (Bausela-Herreras et al., 2019).

When it comes to understanding Executive functions (EF) in children, according to Reilly et al. (2022), EF are key predictors of long-term success that develop rapidly in early childhood, but EF’s developmental trajectories from preschool are not fully understood, and how these trajectories differ based on characteristics of children and their families (based on income, ethnicity, urban versus rural environment, etc.) remains to be characterized. These authors found high individual variability in EF trajectories in children depending on their baseline EF performance, such that children with higher EF at preschool (2 to 4 years-old) entry showed relatively steeper growth during preschool compared to low-EF peers, but those differences attenuated by the end of kindergarten (4 to 6 years-old), which makes it necessary to examine these different trajectories in detail in future studies, to better understand the status and potential trajectories of EF in later stages of childhood and early adolescence. Separately, Davidson et al. (2006) found that cognitive flexibility (switching between rules), even with memory demands minimized, showed a longer developmental progression, with 13-year-olds still not at adult levels. Moreover, Duncan (2006) emphasizes the role of socioeconomic status as a differential factor for the development of EF in children at this age. Probably, the best account of developmental trajectories of EF in later childhood was done by Best and Miller (2010), who talk about (1) rapid changes in inhibition from 3 to 5, less rapid from 6 to 8, and more stable since that age (despite the continuation of brain maturation); (2) a linear increase in working memory from ages 4 to 14 and a leveling off between ages 14 and 15 across nearly all tasks examined, and (3) a protracted development of the ability to successfully shift between task sets through adolescence, from preschool-aged children who can handle shifts between simple task sets and older children who later can handle unexpected shifts between increasingly complex task sets. Both behavioral and physiological measures indicate that during adolescence, monitoring of one’s errors is evident, and by middle adolescence, task switching on these complex shift paradigms typically reaches adult-like levels.

In this context, one of the most significant problems in understanding executive functions is the breadth and diversity of criteria used to define them. For example, Zelazo and Müller (2002) distinguished between (1) the ‘cold’ executive function component, more purely cognitive, associated with the dorsolateral prefrontal cortex and, according to Hongwanishkul et al. (2005, p. 618), more likely to me measured by “abstract decontextualized problems” like the task presented in the Wisconsin Card Sorting Test; and (2) the ‘hot’ executive function component, in charge of regulating aspects that are associated with a relevant emotional component (Mehsen et al., 2021), associated with the ventromedial-prefrontal cortex areas, and more likely to be measured by tasks that involve the regulation of affect and motivation. Since the existence of pure processes is rare, the usual understanding is that EF display a joint and synchronized job between both systems in order to achieve the most efficient result in each situation (Best and Miller, 2010).

When it comes to their assessment, EF share the same problems and challenges as other cognitive functions. Rabbitt (1997) drew attention to the low test–retest reliability and uncertain construct validity of executive function tests; the difficulties to relate functions to specific neuroanatomical areas or neurophysiological systems; the problem of identifying what ultimately are just tasks demands (such as inhibition, planning, monitoring or control) with different system architectures when in fact could be produced by the same system architecture; or the identification of task performance indices and system performance characteristics as equivalent to statistical constructs such as the general intelligence factor. Separately, Díaz-Orueta et al. (2014) pointed out that classical neuropsychological assessment does not reproduce the wide range of stimuli an individual may encounter in their daily life. More specifically, the classical evaluation environment (e.g., a health care center, an office) is closer to a “lab environment,” does not offer any contextual cues to the patient (as real-life environments do), distractors are minimized or erased, sensory modalities are assessed separately, and environmental noise and temperature are set as stable conditions for everyone. Moreover, classical evaluation tests are conditioned by a floor or ceiling effect, tend to evaluate the information storage in a relatively brief period of time, and demand learning of information that does not have any personal relevance for the patient.

Despite the wide availability of traditional paper-and-pencil tools for the purported assessment of executive functions (Lalonde et al., 2013), these tools may show some patients showing a test performance better than expected (or within normal limits) and yet displaying difficulties with activities of daily living, which makes the prediction of patient’s future behavior on the basis of these assessment tools highly questionable. Bombín et al. (2014) stated that the strategy traditionally followed for the evaluation of executive functions has been its atomization in different cognitive threads, as shown in previous studies by Miyake et al. (2000a,b). However, in clinical practice, the disintegration of a global and complex cognitive process like this into countless related subcomponents is often problematic to grasp performance in executive functions in its entirety (Lezak, 1982; Chan et al., 2008) due to problems associated to measurement of functionality, ecological validity and task-impurity (or the inability of traditional EF tasks to measure EF only and measure EF to its maximum extent -Snyder et al., 2015). Miyake et al. (2000a) recognized that the assessment of executive functions needs to overcome serious problems of conceptualization, measurement, lack of correspondence between anatomical structures and functions (i.e., there is no direct correspondence between “frontal lobes” and EF), task impurity, low reliability of classical tests and construct validity. Subsequently, the tests designed according to this paradigm are often of limited value for clinical procedures (such as diagnosis or rehabilitation plans) due to the poor correspondence with the clinical reality of the patient.

These discrepancies suggest that classical neuropsychological tests may not adequately reproduce the complexity and dynamic nature of real-life situations. To overcome these limitations, latest technological developments such as virtual reality (VR) based neuropsychological assessment tools, may achieve greater accuracy and validity for the assessment of a wide range of cognitive functions, including executive functions (Climent et al., 2014; Kim et al., 2021; Borgnis et al., 2022).

Virtual reality reproduces three-dimensional environments with which the patient interacts dynamically, with a feeling of immersion in the environment similar to the presence and exposure to a real environment. In addition, the presentation of target stimuli, as well as distractors or other variables, can be systematically controlled. Likewise, more consistent and precise answers can be obtained, as well as a detailed analysis of them (Camacho-Conde and Climent, 2022; Kusi-Mensah et al., 2022; Silva et al., 2022). Kim et al. (2021) describes that fully immersive virtual reality (VR) as a promising resource, not only necessary to overcome the existing limitation of neuropsychological tests, but also for the development of tailored treatments for EF within activities of daily living (ADLs) due to its high ecological validity, which is in line with recent reviews on the topic (Borgnis et al., 2022).

Subsequently, in order to overcome the existing limitations and develop on the potential provided by the latest Virtual Reality based technologies, the aim of this study was to obtain normative data for a new developed VR based neuropsychological test, the Ice Cream VR test, on a population of children between 8 and 16 years old. Ice Cream is a VR test designed to evaluate executive functions including Processing speed, Working memory, Planning, Learning, Cognitive flexibility, interference and Perseverations, and help clinicians complement the information included in the diagnosis and subsequent follow-up of any disorder that affects these parameters. Prior to the Ice-Cream test, one of the best examples of VR based tests for EF is the Jansari assessment of Executive Functions for Children (JEF-C) by Gilboa et al. (2019), a non-immersive computerized assessment of executive functions, which presented promising results for children and adolescents with acquired brain injury with a complex task that appeared to be both playful as well as sensitive and ecologically valid. Similarly, Ice Cream [like other VR Tests such as AULA (Iriarte et al., 2016) or AQUARIUM (Climent et al., 2021)] shows the advantage of being presented as a VR “game,” thus facilitating the initial predisposal of children and adolescents to the evaluation. In previous studies, Iriarte et al. (2016) found that the game-like scenario provided by AULA VR-based neuropsychological test was reported as a motivational asset for children and adolescents when faced with the cognitive testing. According to Lumsden et al. (2016), careful application of gamification can provide a way to develop engaging and yet scientifically valid cognitive assessments. More recently, Ferreira-Brito et al. (2019) found that narrative context was the main used gamification feature used in video games used for cognitive assessment, as it has no association with player’s performance, but instead helps contextualize and add meaning to the test’s main activity, inspiring motivation and long-term willingness toward tasks that may be perceived as boring and repetitive in its non-gamified version. In this context, hence, it is important to highlight that although, *a priori*, the Ice Cream VR test may seem like a playful activity, it is a really intense cognitive exercise but initially, much better perceived and more stimulating for the subjects than the classic paper and pencil tests.

The following Method section will present a description of the normative sample and the Ice-Cream VR test variables and measures. Due to the complexity of the test, for the Results section we have moved beyond the mere description of normative data. Consequently, the Results section will provide a detailed statistical rationale of the results for the total sample, separate distributions by sex and age with associated normality and homoscedasticity analyses, a cluster analysis by age, an in-depth analysis of the validity and reliability of the scales, a confirmatory factor analysis that evidences the main variables measured by the Ice-Cream VR test and a detailed analysis of the test reliability and internal consistency. With this structure, the current study aims to both present normative groups for the general population for the Ice-Cream VR test as well as provide further understanding on the construct validity and scales contained in the test.

## 2. Methods

### 2.1. Participants

The normative sample comprised a total number of *n* = 821 participants (49% female), with an age range of 8 to 16 years old, recruited across nine different testing sites in Spain. Inclusion criteria required no neurological pathology, sensory alterations or other type of condition that may limit the use of the virtual reality devices necessary for the evaluation, and being native in Spanish as it was the main language for the assessment tool in this normative study. Table 1 shows the distribution by sex and age for the normative sample.

**Table 1:** Sample distribution by age and sex.

Age	Sex	Total	Percentage
8	Female	34	2.74
8	Male	32	2.58
9	Female	56	4.52
9	Male	70	5.65
10	Female	63	5.08
10	Male	65	5.24
11	Female	45	3.63
11	Male	58	4.68
12	Female	48	3.87
12	Male	38	3.06
13	Female	37	2.98
13	Male	39	3.15
14	Female	53	4.27
14	Male	57	4.60
15	Female	49	3.95
15	Male	39	3.15
16	Female	15	1.21
16	Male	23	1.85

The sample size is 821.

The target number of participants to be included in the study in order to ensure representativeness of the general population in Spain was done according to three criteria: age, gender and educational level. The target numbers were estimated according to the ratios obtained for these three criteria from the data of the census from the National Institute of Statistics in Spain for the year 2016 (the latest available up to date).

The sample size estimation was performed with the assistance of two psychometricians, according to practical feasibility criteria and considering the cost–benefit balance (Prieto-Valiente and Herranz, 2004). A minimum of 400 people whose sociodemographic characteristics were representative of the general Spanish population was recommended. Following a procedure recommended by the psychometricians involved in the study, as it was done previously in other normative studies (Iriarte et al., 2016, for example), no specific evaluations were previously performed to exclude children with potential psychiatric disorders or other neurodevelopmental disorders. The rationale for this was that, in order to ensure a recruitment from the general population as representative as possible, no disorder-specific exclusion criteria would be set; so that any potential prevalence of psychiatric or neurodevelopmental disorders in the normative sample would be a fair representation of that same prevalence in the general population.

The administration of the test was carried out by evaluators recruited by the company Giunti-Nesplora, developer of the test, trained for the use of the VR equipment and the administration of the Ice Cream VR test. Data collection was conducted in nine different cities across Spain in order to ensure geographical representativeness of the sample. Moreover, a questionnaire collecting socio-demographic data from participants (e.g., educational level, occupation, languages spoken, etc.) was administered.

Prior to the study, and in order to comply with ethical guidelines, signed informed consent forms were obtained from participants (only for those who were already 16 years old, according to the Spanish legislation) and from their parents or guardians (for the majority of participants under 16). The Ethical Committee approved the study and the data collection protocol for Research with Human Beings. The study was carried out in accordance with the Code of Ethics of the World Medical Association (Declaration of Helsinki) for experiments involving humans.

### 2.2. Measure

Nesplora Ice Cream is a test oriented to assess executive functions by simultaneously measuring learning, planning, attention, working memory, cognitive flexibility, processing speed, interference and perseverations. It was designed as a test to support the diagnosis and a measure of efficacy and follow up for treatments targeting learning and other cognitive problems. As the name suggests, the test takes place in a virtual ice cream shop where the testee must attend to a series of customers, while observing a number of rules or criteria, and serve them the ice creams they ask for.

The task is performed in an environment that simulates an ice cream shop. The testee is given a set of VR glasses with movement sensors that allow them to see and hear what happens in that VR environment, thus immersing the individual in the virtual ice cream shop environment. All task instructions are presented on an auditory basis. The perspective places the subject within the counter, oriented to the customers. Head movements are captured by the headset and the software updates the scene, giving the subject the impression of actually being in the virtual environment. The subject then begins by performing a usability task that will help them get familiarized with the environment and the task. It is understood that the cashier is the one telling the individual what to do (i.e., the testee listens to an audio speech with instructions). Here, they must complete the task by pressing a button when pointing to certain objects indicated by the cashier (i.e., the ice cream making machine, a paper basket, the recipe book, a phone and a clock).

Once the usability task is done, the voice of the cashier appears again saying that the boss will call to provide a series of rules or criteria that the testee must strictly adhere to when it comes to serving the customers, as follows: “*You’ll be working at the ice cream shop for a while. Customers come in groups of four and you must serve them following your boss’s orders. Call your boss and he’ll tell you his priorities to serve customers. Click on the phone to call him*.” Then, the individual must point to the phone and push the button to make a call. The boss will explain the instructions “*First you have to serve the surf students. They come with a neoprene surf suit, and they leave the floor soaking wet. Then, the people in suits, who are from a nearby company and usually in a hurry. They carry an identification badge on their chests. Third, serve the volunteers who are cleaning the beach. They’re wearing reflective vests. And within this order, always serve those who have a ticket first, as they have already paid for their ice cream. For example, if there are two people wearing suits, serve the one with the ticket first. If you do not remember your boss’s priorities, you can call him on the phone whenever there aren’t any customers in the shop*.”

**Figure 1:**
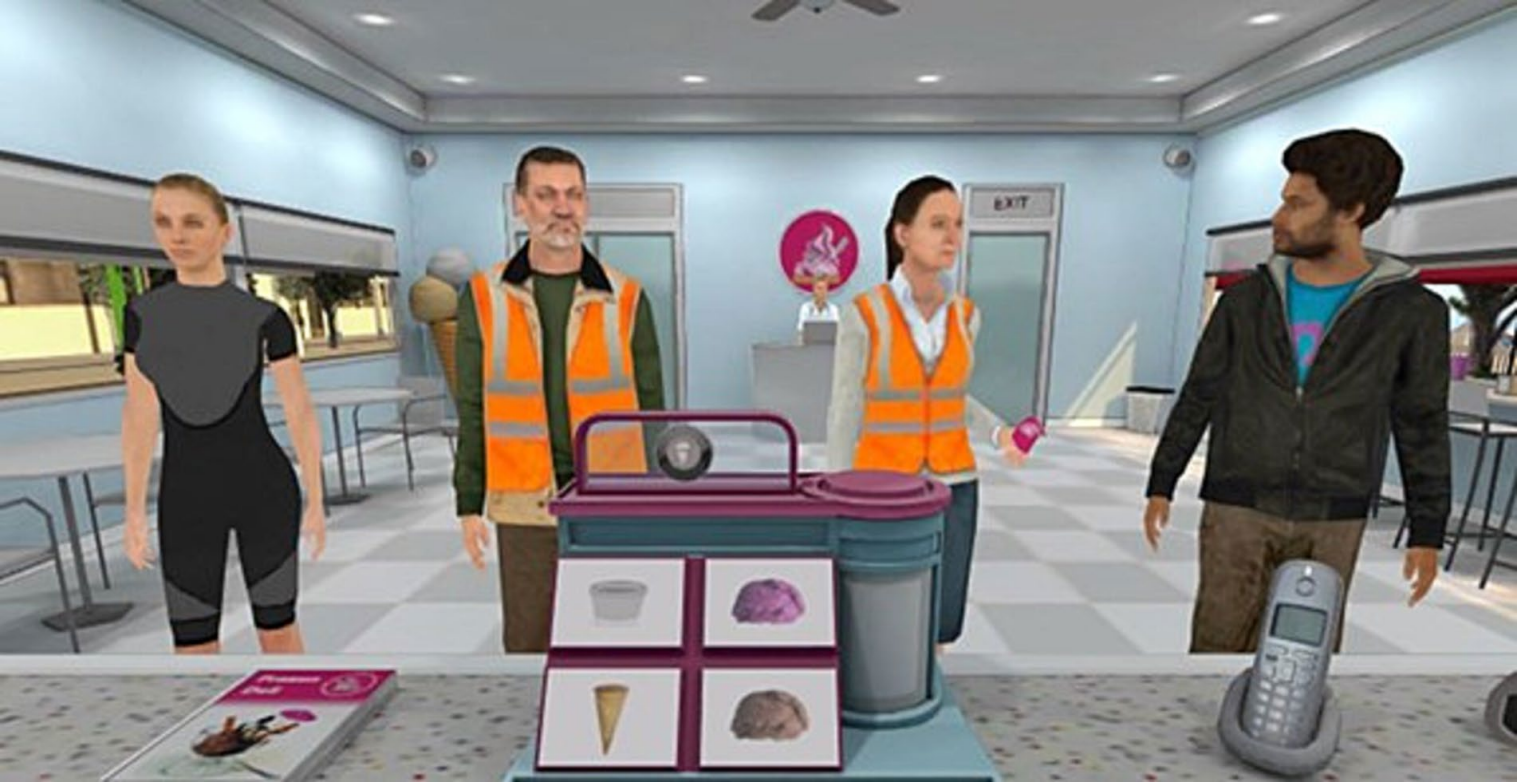
Screenshot of Nesplora Ice Cream test, from the test taker perspective. Reproduced with permission from Giunti-Nesplora SL.

After this, there will be a trial to test the different instructions set, the assignment of shifts according to what clothes clients wear and the different ice cream recipes. The training makes the participant fail in order to show them how to throw the wrong ice cream in the bin. The test registers every click as well as every response time and inter-click latencies between different events (i.e., every click made over the avatars of the customers, the buttons on the ice cream making machine, or other incorrect objects during this training trial). During the training the book is shown 4 times for the same amount of time so that all participants are exposed in the same way to be able to learn the recipes equally (see Figure 1).

Then, the actual test will start with the first group of four customers. With each group of customers (14 in total during the test), the test taker must:

1. click on the individual customers in the right order (according to the instructions given by the boss) in order to set their order
2. turn on the ice cream making machine.
3. click on the individual customer who must be the first according to the established order,
4. prepare the ice cream requested by each customer (ice cream #1, 2, 3 or 4 from the recipe book), if possible, without looking at the recipe book,
5. give each ice cream to the right customer.

Overall, the performance in the Ice Cream Seller Test can be divided into three general tasks: (1) Planning: give the customers their turn according to previously specified rules or criteria, (2) Learning, part A (working memory): serve the ice creams to the customers while consulting the recipe book as little as possible, (3) Learning, part B (cognitive flexibility): serve the ice creams to the customers while consulting a new modified version of the recipe book as little as possible. In both parts A and B the test works with the same structure, environment and task. However, when the individual is halfway doing the test, the initially learnt series of ice creams changes, and a new set of ice cream variants need to be learnt to perform correctly in the second half of the test, thus intending to demand some cognitive flexibility from the subject. The planning and the preferences set to attend the customers are thus maintained, while the ice cream variant change implies to unlearn some cues and relearn a new different set of cues.

In terms of variables measured, the test captures different performance measures across the tasks. In the Planning task, the test collects information on processing speed and rule learning (correct customer order designation, correct ice cream delivery). In the second task (learning, part A, working memory), there are measures of processing speed and learning potential. In the third task (learning, part B, cognitive flexibility) measures on processing speed, interference, perseverations and switching are collected. Overall composite indices of planning, working memory and cognitive flexibility are provided at the end.

Thus, the indices provided in the report for planning include:

- Planning: the number of assignments of customers performed in the right order.
- Assignment time: time required to perform the assignment, regardless of being a correct or incorrect assignment.
- Cognitive load: a measure of how the increasing difficulty of the test affects planning. It is calculated by comparing errors of the first half versus the second half of the test.
- Fatigue: It is measured by comparing time to complete the second half of the test versus time to complete the first half.
- Prospective planning: ability to remember to turn on the ice cream making machine. The subject must do this at the beginning of each of the 14 rounds with customers.
- Coherence indicator: the subject performs the task as planned, even if it was planned wrong according to the given instructions.
- Impulsivity: when the subject clicks on the phone while there are customers in the shop.
- Incorrect assignments: the subject makes the right ice cream but gives it to the wrong customer. It is associated with poor attention or immediate memory.

Second, the indices provided in the report for working memory will include:

- Correct services: number of ice creams correctly sold.
- Consultations: number of times the subject had to consult the recipe book or call the boss.
- Net correct answers: Number of clients correctly assigned and served without any consultations. It indicates the subject’s ability to process, encode and keep the information.
- Time of service: time required by the subject to perform each particular action.

Finally, the indices provided in the report for cognitive flexibility are:

- Interference: it measures to what extent the learning and practice with the first recipe books interferes with the learning of the new set of ice cream variants (i.e., the new recipe book). Here, the clinician must judge whether an outstanding performance in the second half of the test, with the new recipe book, reflects either cognitive flexibility or, on the contrary, reflects a new learning (if the performance in the first half with the first recipe book was poor).
- Switching: it refers to the ability to perform with the new recipe book. It takes into account the performance in the two last trials with the first recipe book, and the two first trials with the new recipe book.
- Perseverations: it indicates the number of wrong items of the second half of the test that would be correct in the first half (with the initial recipe book).

It is important to mention that the Ice Cream VR test produces more than 1867 variables with the information generated in the evaluation. Of all these variables, a total of 1,055 were selected for what will constitute the clinical report of the test to be used in the future with clinical samples. This selection has been based on clinical criteria and ease of interpretation. The rest of the variables may be used in the future either to prepare other types of reports or to complement the existing clinical report. Therefore, the results shown in this section correspond to the main variables that appear in the report, which were selected based on their expected clinical utility. Supplementary Tables 1, 2 show the main final variables used in the clinical report and their corresponding abbreviations.

## 3. Results

In this section we present the results of the test administration carried out in Spain on people aged between 8 and 16 years old for the obtention of normative data for the Ice Cream VR Test.

The variables taken for each of the subtests to determine the scales were as follows. These variables have been selected from the set of variables under psychological criteria and according to what is to be measured in each subtest, and these criteria were on the basis of the statistical procedures (i.e., cluster analyses and confirmatory factor analysis) presented, respectively, in subsections 3.3 and 3.6 of this Results section. The scales and variables they comprise are presented below.

Planning:

- Number of shifts correctly assigned in Part 1.
- Number of shifts correctly assigned in Part 2.
- Learning potential to identify whether the customer wears a neoprene suit or not, (measured at Round 13).
- Learning potential when it comes to assign the right order to the customers.

Learning:

- Number of total correct ice creams delivered correctly without looking at the recipe book on Part 1 rounds.
- Number of correct #1 ice creams delivered without looking at the recipe book in Part 1 rounds.
- Learning potential in relation to making ice cream #1 correctly.

Flexibility:

- Number of total correct ice creams delivered correctly without looking at the recipe book on Part 2 rounds.
- Number of correct #1 ice creams delivered without looking at the recipe book in Part 2.
- Number of perseverations when making the ice creams in Part 2.
- Learning potential in terms of flexibility when making ice cream #4 in Part 2 (which was ice cream #1 in Part 1).
- Learning potential in terms of flexibility when making ice cream #1 in Part 2 (which is different from ice cream #1 in Part 1).

### 3.1. Results for the total sample

Next, we describe the variables for the total sample. Secondly, the differences according to sex and age found in the normative sample are shown. Third, the normative groups obtained, and the homoscedasticity and normality analysis are described. Finally, the reliability of the Nesplora Ice Cream test scales, a confirmatory factor analysis, and test reliability and internal consistency will be presented.

Table 2 presents the overall results for the total sample.

**Table 2:** Description of variable results for the total sample (*n* = 821).

	Mean	SD	Q1	Median	Q3	Max	Skew	Kurtosis
Number of shifts correctly assigned in Part 1	4.68	2.26	3	6	7	7	−0.51	−1.13
Number of shifts correctly assigned in Part 2	4.63	2.56	2	6	7	7	−0.64	−1.18
Learning potential to identify whether the customer wears a neoprene suit	132.01	98.56	18	146	242	242	−0.14	−1.63
Learning potential when it comes to assign the right order to the customers	160.82	137.27	0	189	288	341	0.04	−1.66
Number of total correct ice creams delivered correctly without looking at the recipe book on Part 1 rounds	24.01	5.74	23	26	28	28	−2.27	5.39
Number of correct #1 ice creams delivered without looking at the recipe book in Part 1 rounds.	10.58	2.45	10	12	12	12	−2.46	6.46
Number of correct #1 ice creams delivered without looking at the recipe book in Part 2.	8.27	2.24	7	9	10	10	−1.64	2.43
Number of correct #1 ice creams delivered without looking at the recipe book in Part 2.	21.09	6.23	18	23	26	28	−1.23	1.09
Learning potential in relation to making ice cream #1 correctly	114.50	59.86	74	138	164	164	−0.79	−0.89
Learning potential in terms of flexibility when making ice cream #4 in Part 2 (which was ice cream #1 in Part 1)	69.75	55.52	9	74	121	147	0.08	−1.48
Number of perseverations when making the ice creams in Part 2	1.32	1.82	0	1	2	16	2.22	8.00
Learning potential in terms of flexibility when making ice cream #1 in Part 2 (which is different from ice cream #1 in Part 1)	59.40	51.24	4	58	125	125	0.18	−1.62

The sample size is 821 and the minimum for each variable is 0.

As can be observed after studying the frequencies of the values obtained from the sample, most of the variables are distributed asymmetrically. Since the analysis of samples that do not have a normal distribution becomes a problem in common statistical parametric tests that assume normality in the data, specific procedures-methods that assume *de facto* that type of distribution have been used (Brown and Forsythe, 1974a), instead of attempting one of the following transformations: logarithmic, square root, or inverse. To test the normality of the sample according to sex, we tested whether or not the data set fits a normal distribution. For this purpose, a data Energy test was performed (Székely and Rizzo, 2017). Data energy is the value of a real function of distances between data in metric spaces. The name energy is derived from Newton’s gravitational potential energy, which is also a function of distances between physical objects. One of the advantages of working with energy functions (energy statistics) is that even if the data are complex objects, such as functions or graphs, we can use their real-valued distances for inference. This type of test has been used in studies on multivariate normality obtaining high accuracy in the results. The direct connection between energy and mind/observations/ data is a counterpart of the equivalence of energy and matter/mass in the equation: Albert Einstein’s *E* = mc^2^.

For this reason of asymmetry, the following section will show different results for gender and age groups, each of them followed by an analysis of normality and homoscedasticity.

### 3.2. Distribution by sex with associated normality and homoscedasticity analyses

Table 3 shows the descriptive results for the male participants of the normative sample (*n* = 421).

**Table 3:** Descriptive data for each variable with respect to sex: male.

Variable	Mean	Std. dev	Median	Max	25th	75th	Skew	Kurtosis
Number of shifts correctly assigned in Part 1	4.74	2.27	6	7	3	7	−0.54	−1.12
Number of shifts correctly assigned in Part 2	4.73	2.52	6	7	2	7	−0.72	−1.04
Learning potential to identify whether the customer wears a neoprene suit	130.88	97.35	146	242	18	242	−0.13	−1.60
Learning potential when it comes to assign the right order to the customers	159.03	136.66	153	341	10	288	0.06	−1.66
Number of total correct ice creams delivered correctly without looking at the recipe book on Part 1 rounds	24.00	5.82	26	28	23	28	−2.14	4.63
Number of correct #1 ice creams delivered without looking at the recipe book in Part 1 rounds.	10.56	2.49	12	12	10	12	−2.38	5.81
Number of correct #1 ice creams delivered without looking at the recipe book in Part 2.	114.13	60.00	138	164	74	164	−0.78	−0.91
Number of correct #1 ice creams delivered without looking at the recipe book in Part 2.	21.19	5.96	23	28	18	26	−1.18	1.05
Learning potential in relation to making ice cream #1 correctly	8.36	2.11	9	10	7	10	−1.58	2.39
Learning potential in terms of flexibility when making ice cream #4 in Part 2 (which was ice cream #1 in Part 1)	1.42	1.98	1.	16	0	2	2.36	9.00
Number of perseverations when making the ice creams in Part 2	69.71	55.70	74	147	9	121	0.07	−1.50
Learning potential in terms of flexibility when making ice cream #1 in Part 2 (which is different from ice cream #1 in Part 1)	58.78	50.96	58	125	4	125	0.19	−1.61

The sample size is 421 and the minimum for each variable is 0.

In order to verify normality for each variable considering sex, the non-parametric Anderson-Darling test was used (Marsaglia and Marsaglia, 2004). This test is a modification of the Kolmogorov–Smirnov test (Shapiro et al., 1968) where more weight is given to the tails. It uses a specific distribution to calculate the critical values. This has the advantage of allowing a more sensitive test and the disadvantage that critical values must be calculated for each distribution. The starting hypotheses are:

*H0*: the data are from a normal distribution.

*H1*: data are not from a normal distribution.

Applying an Anderson–Darling Test on the subset of data pertaining to the male sex for the selected variables (listed in Table 3) non-normality was obtained with a *p*-value under 0.00 (df = 12.19).

Separately, Table 4 shows the descriptive results for female participants of the normative sample (*n* = 400).

**Table 4:** Descriptive data for each variable with respect to sex: female.

Variable	Mean	Std. Dev	Median	Max	25th	75th	Skew	Kurtosis
Number of shifts correctly assigned in Part 1	4.61	2.25	5	7	3	7	−0.47	−1.14
Number of shifts correctly assigned in Part 2	4.53	2.62	6	7	2	7	−0.56	−1.31
Learning potential to identify whether the customer wears a neoprene suit	133.21	99.92	146	242	32.25	242	−0.15	−1.67
Learning potential when it comes to assign the right order to the customers	162.71	138.07	189	341	0	288	0.02	−1.66
Number of total correct ice creams delivered correctly without looking at the recipe book on Part 1 rounds	24.01	5.66	26	28	23	28	−2.40	6.21
Number of correct #1 ice creams delivered without looking at the recipe book in Part 1 rounds.	10.60	2.40	12	12	10	12	−2.56	7.17
Number of correct #1 ice creams delivered without looking at the recipe book in Part 2.	114.88	59.77	151	164	74	164	−0.81	−0.87
Number of correct #1 ice creams delivered without looking at the recipe book in Part 2.	20.98	6.51	23	28	18	26	−1.24	1.03
Learning potential in relation to making ice cream #1 correctly	8.18	2.36	9	10	7	10	−1.65	2.25
Learning potential in terms of flexibility when making ice cream #4 in Part 2 (which was ice cream #1 in Part 1)	1.20	1.62	1	9	0	2	1.79	3.66
Number of perseverations when making the ice creams in Part 2	69.81	55.40	74	147	9	121	0.10	−1.47
Learning potential in terms of flexibility when making ice cream #1 in Part 2 (which is different from ice cream #1 in Part 1)	60.04	51.58	58	125	4	125	0.16	−1.64

The sample size is 400 and the minimum for each variable is 0.

Similarly, an Anderson-Darling Test was applied on the subset of data belonging to the female sex for the selected variables (see Table 4) and non-normality was obtained with a *p*-value below 0.00 (df = 11.83).

The assumption of homogeneity of variances (homoscedasticity) considers that the variance does not vary for the different values of a variable belonging to different groups. That is, as a null hypothesis, it considers that the variance is equal between groups and as an alternative hypothesis that it is not.

As many of the variables follow an asymmetric distribution, we have chosen to use the Brown–Forsythe test (Brown and Forsythe, 1974b) whose centrality statistic is the median, which offers good robustness to many types of non-normal data while retaining good statistical power. This test makes it possible to test for equality of variance in 2 or more populations without the need for the size of the groups to always be the same. Table 5 shows the homoscedasticity results with respect to sex.

**Table 5:** Homoscedasticity with respect to sex.

Variable	Brown–Forsythe Statistic	Denom *df*	*p*-value
Number of shifts correctly assigned in Part 1	0.743	817.305	0.389
Number of shifts correctly assigned in Part 2	1.264	812.422	0.261
Learning potential to identify whether the customer wears a neoprene suit	0.114	814.136	0.736
Learning potential when it comes to assign the right order to the customers	0.147	815.914	0.701
Number of total correct ice creams delivered correctly without looking at the recipe book on Part 1 rounds	0	818.511	0.985
Number of correct #1 ice creams delivered without looking at the recipe book in Part 1 rounds.	0.06	818.882	0.806
Learning potential in relation to making ice cream #1 correctly	0.033	817.163	0.857
Number of total correct ice creams delivered correctly without looking at the recipe book on Part 2 rounds	0.254	803.446	0.615
Number of correct #1 ice creams delivered without looking at the recipe book in Part 2	1.377	797.386	0.241
Number of perseverations when making the ice creams in Part 2	2.917	801.455	0.088
Learning potential in terms of flexibility when making ice cream #4 in Part 2 (which was ice cream #1 in Part 1)	0.001	817.276	0.98
Learning potential in terms of flexibility when making ice cream #1 in Part 2 (which is different from ice cream #1 in Part 1)	0.123	815.718	0.726

All variables have a “num df” = 1.

As can be seen in Table 5, the null hypothesis is accepted for all the variables presented, hence, the variance of all the variables is equal for male and female participants. As the null hypothesis is accepted for the variables of the planning, learning and flexibility subtests, the cluster analysis will not differentiate between women and men, implying that there is no need to present separate normative data groups based on gender.

### 3.3. Cluster analysis by age

**Figure 2:**
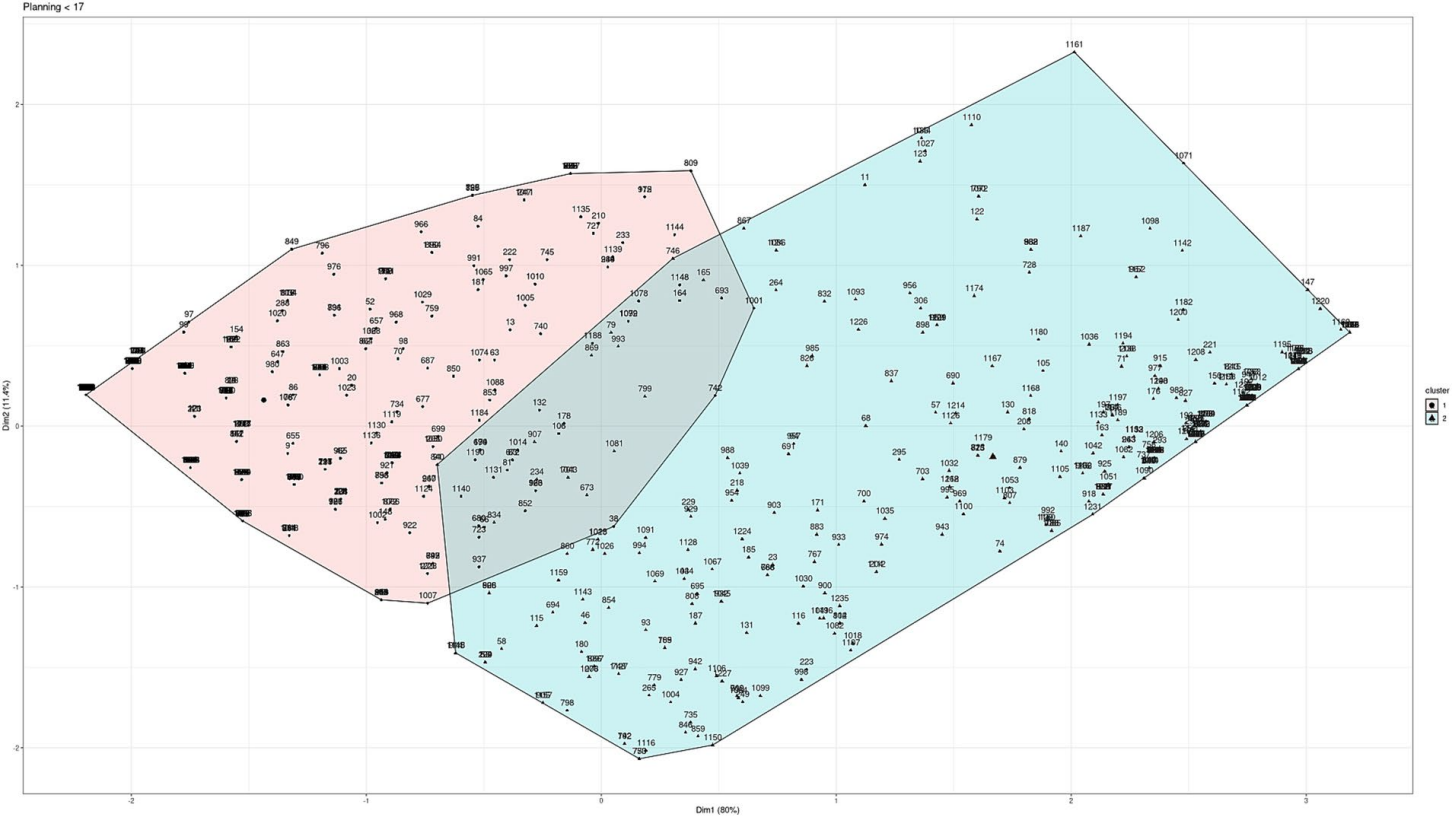
Cluster analysis for planning.

**Figure 3:**
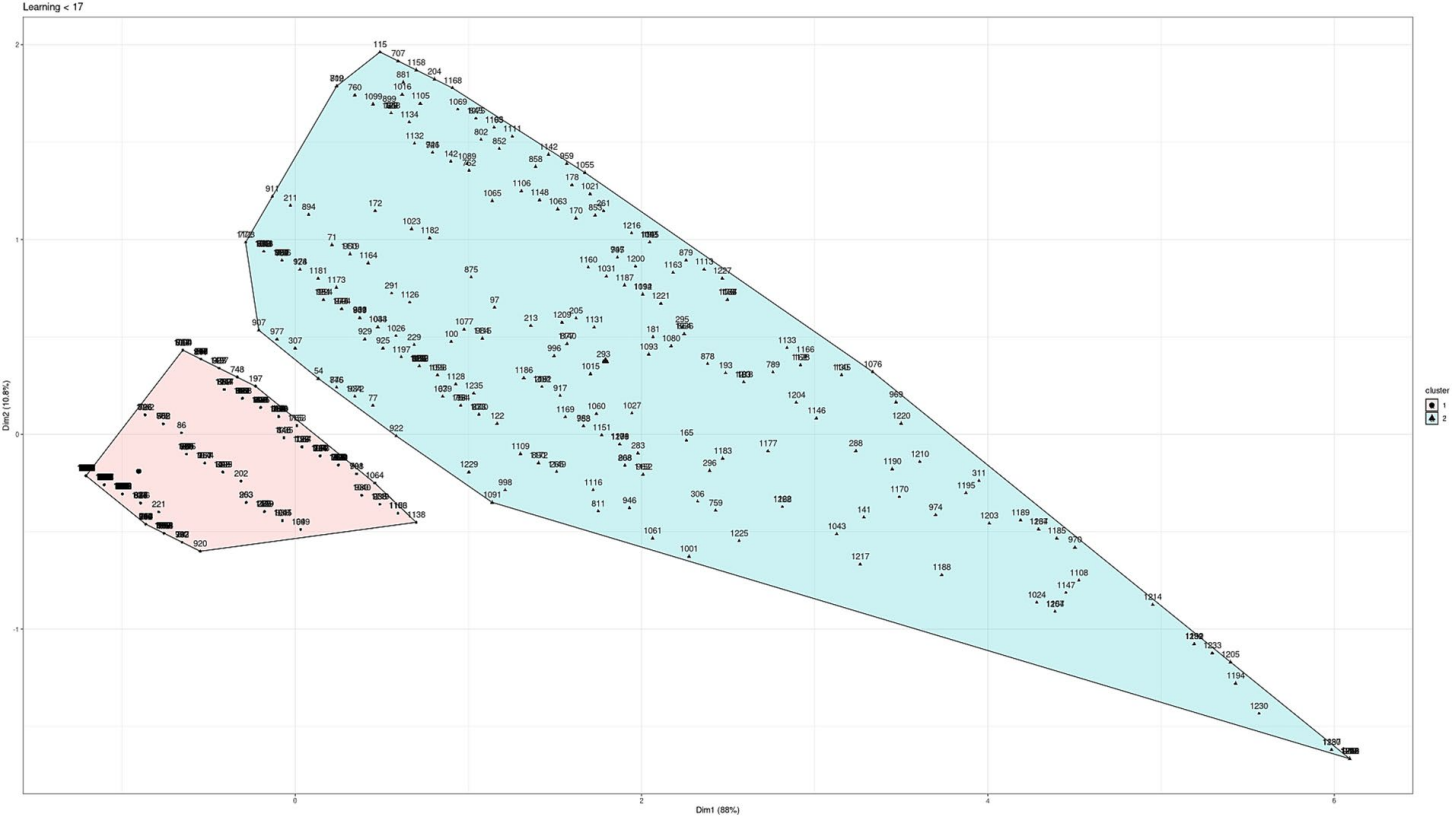
Cluster analysis for learning.

**Figure 4:**
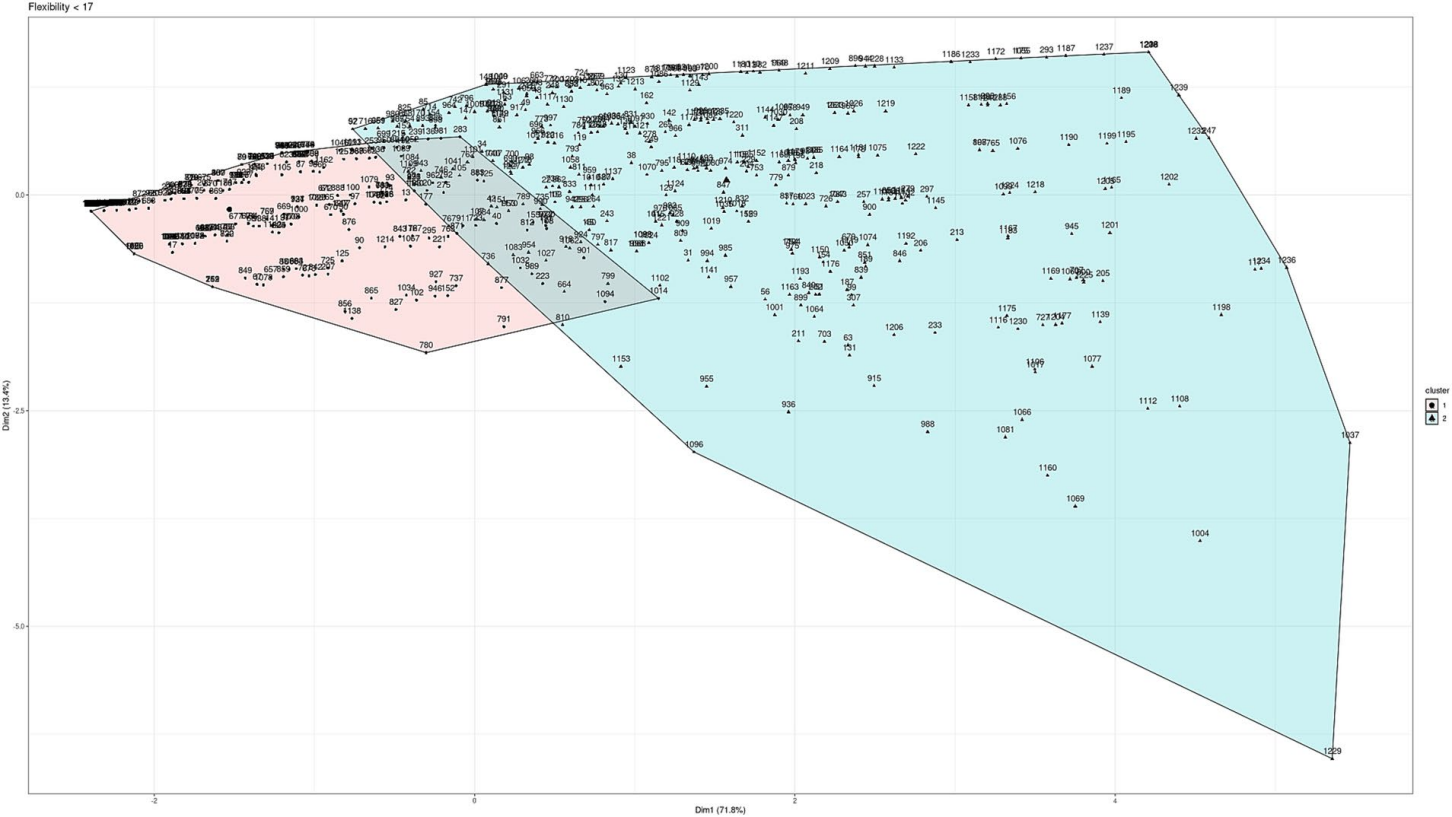
Cluster analysis for cognitive flexibility.

To determine the scales according to age, different clustering techniques were used (“hierarchical,” “kmeans,” “diana,” “model,” “pam,” “clara,” “agnes”). Testing with different techniques allows us to work with the technique that presents greater robustness and greater clarification of the groups according to the data we are working with. Between the ages of 8 and 16, three groups have been established for the scales according to age for the three subtests: planning (8–11 and 12–16), learning (8–9 and 10–16), and flexibility (8–11 and 12–16), as shown in Figures 2–4 (and associated Table 6). For planning, the division of 11 years old showed a high proximity of values (47 vs. 56), hence, it was decided to build a cluster between 8 and 11 years old and thus match the groups obtained for flexibility in a more consistent way. It can be seen that the two main dimensions generated explain more than 85% of the subjects in the sample.

**Table 6:** Clustering with respect to age (<17) and subtest: planning, learning, and cognitive flexibility.

Scale	Age									
Planning	Age	8	9	10	11	12	13	14	15	16
		10	37	47	**56**	**52**	**52**	**87**	**70**	**30**
		**56**	**89**	**81**	47	34	24	23	18	8
Learning	Age	8	9	10	11	12	13	14	15	16
		21	51	**71**	**68**	**62**	**56**	**100**	**80**	**37**
		**45**	**75**	57	35	24	20	10	8	1
Cognitive flexibility	Age	8	9	10	11	12	13	14	15	16
		9	35	50	44	**52**	**55**	**81**	**63**	**27**
		**57**	**91**	**78**	**59**	34	21	29	25	11

Bold values show the highest value.

### 3.4. Distribution by age with associated normality and homoscedasticity analyses

Data from 821 subjects were initially analyzed and 3 age groups were identified to obtain the scales (8–9, 10–11, 12–16). Table 7 shows the sample distribution according to these clustered age groups.

**Table 7:** Sample distribution by clustered age groups.

Years	Sex	Total	Percentage per age cluster
08–09	Female	90	46.88
08–09	Male	102	53.12
10–11	Female	108	46.75
10–11	Male	123	53.25
12–16	Female	202	50.75
12–16	Male	196	49.25

The sample size is 821.

To check the normality for the normative groups, the same test has been used, an Energy Test, used in the contrast of the normality of the sample according to sex. Also in this case we will test whether or not the data set conforms to a normal distribution.

#### 3.4.1. Planning

Normality for Planning subtest for the under 17 age scale is shown below. Table 8 shows the data for the 8 to 11 years old Planning cluster. No variable shows a normal distribution.

**Table 8:** Planning variable with respect to age 8–11: descriptives and normality tests (Anderson-Darling test and multivariate normality *E*-statistic test).

Variable	Mean	Std. Dev	Median	Max	25th	75th	Skew	Kurtosis	df (A–D test)	df (*E*-test)
Number of shifts correctly assigned in Part 1	3.78	2.27	3	7	2	6	0.05	−1.29	11.5270^*^	13.75^*^
Number of shifts correctly assigned in Part 2	3.72	2.61	4	7	1	6	−0.07	−1.56	19.0935^*^	
Learning potential to identify whether the customer wears a neoprene suit	96.73	94.31	65	242	5	192	0.43	−1.40	26.5873^*^	
Learning potential when it comes to assign the right order to the customers	105.65	124.36	24	341	0	236	0.71	−1.13	38.4602^*^	

The sample size is 423 and the minimum of each variable is 0.

* All variables show “NOT normality” with a *p* < 0.001.

Table 9 shows the data for the 12 to 16 years old Planning cluster. No variable shows a normal distribution.

**Table 9:** Planning variable with respect to age 12–16: descriptives and normality tests (Anderson-Darling test and multivariate normality *E*-statistic test).

Variable	Mean	Std. dev	Median	Max	25th	75th	Skew	Kurtosis	df (A–D test)	df (*E*-test)
Number of shifts correctly assigned in Part 1	5.62	1.83	6	7	5	7	−1.25	0.42	38.7009^*^	39.31^*^
Number of shifts correctly assigned in Part 2	5.60	2.12	7	7	5	7	−1.51	0.91	52.4739^*^	
Learning potential to identify whether the customer wears a neoprene suit	169.52	88.72	242	242	102	242	−0.79	−0.91	38.3730^*^	
Learning potential when it comes to assign the right order to the customers	219.46	125.67	288	341	120	341	−0.65	−1.07	24.5681^*^	

The sample size is 398 and the minimum of each variable is 0.

* All variables show “NOT normality” with a *p* < 0.001.

#### 3.4.2. Learning

Normality for Learning subtest for the under 17 age scale is shown below. Table 10 shows the data for the 8 to 9 years old Learning cluster. No variable shows a normal distribution.

**Table 10:** Learning variable with respect to age 8–9: descriptives and normality tests (Anderson-Darling test and multivariate normality *E*-statistic test).

Variable	Mean	Std. dev	Median	Max	25th	75th	Skew	Kurtosis	df (A–D test)	df (*E*-test)
Number of total correct ice creams delivered correctly without looking at the recipe book on Part 1 rounds	19.29	7.88	22	28	16	25	−1.09	0.24	8.0861^*^	7.15^*^
Number of correct #1 ice creams delivered without looking at the recipe book in Part 1 rounds	8.64	3.47	10	12	7	11	−1.19	0.45	10.4668^*^	
Learning potential in relation to making ice cream #1 correctly	71.95	61.42	74	164	9	114	0.25	−1.41	8.1849^*^	

The sample size is 192 and the minimum of each variable is 0.

* All variables show “NOT normality” with a *p* < 0.001.

Table 11 shows the data for the 10 to 16 years old Learning cluster. No variable shows a normal distribution.

**Table 11:** Learning variable with respect to age 10–16: descriptives and normality tests (Anderson-Darling test and multivariate normality *E*-statistic test).

Variable	Mean	Std. dev	Median	Max	25th	75th	Skew	Kurtosis	df (A–D test)	df (*E*-test)
Number of total correct ice creams delivered correctly without looking at the recipe book on Part 1 rounds	25.45	3.91	27	28	25	28	−2.80	10.30	63.3906^*^	107.51^*^
Number of correct #1 ice creams delivered without looking at the recipe book in Part 1 rounds	11.17	1.62	12	12	11	12	−3.08	12.69	96.7260^*^	
Learning potential in relation to making ice cream #1 correctly	127.48	53.00	164	164	114	164	−1.22	0.12	77.8759^*^	

The sample size is 629 and the minimum of each variable is 0.

* All variables show “NOT normality” with a *p* < 0.001.

#### 3.4.3. Flexibility

Finally, normality for Flexibility subtest for the under 17 age scale is shown below. Table 12 shows the data for the 8 to 11 years old cluster. No variable shows a normal distribution.

**Table 12:** Flexibility variable with respect to age 8–11: descriptives and normality tests (Anderson-Darling test and multivariate normality *E-*statistic test).

Variable	Mean	Std. dev	Median	Max	25th	75th	Skew	Kurtosis	df (A–D test)	df (*E*-test)
Number of total correct ice creams delivered correctly without looking at the recipe book on Part 2 rounds	18.70	6.58	20	28	15	23.50	−0.84	0.11	7.6019^*^	12.71^*^
Number of correct #1 ice creams delivered without looking at the recipe book in Part 2	7.58	2.49	8	10	6	10	−1.20	0.90	18.9877^*^	
Number of perseverations when making the ice creams in Part 2	1.77	2.10	1	16	0	3	1.99	6.43	23.5434^*^	
Learning potential in terms of flexibility when making ice cream #4 in Part 2 (which was ice cream #1 in Part 1)	47.69	50.46	36	147	0	97	0.71	−0.87	24.7935^*^	
Learning potential in terms of flexibility when making ice cream #1 in Part 2 (which is different from ice cream #1 in Part 1)	40.74	46.50	19	125	0	77	0.83	−0.83	34.4003^*^	

The sample size is 423 and the minimum of each variable is 0.

* All variables show “NOT normality” with a *p* < 0.001.

Table 13 shows the data for the 12 to 16 years old Planning cluster. No variable shows a normal distribution.

**Table 13:** Flexibility variable with respect to age 12–16: descriptives and normality tests (Anderson-Darling test and multivariate normality *E*-statistic test).

Variable	Mean	Std. dev	Median	Max	25th	75th	Skew	Kurtosis	df (A–D test)	df (*E*-test)
Number of total correct ice creams delivered correctly without looking at the recipe book on Part 2 rounds	23.62	4.65	25	28	22	27	−1.93	4.67	20.9735^*^	38.97^*^
Number of correct #1 ice creams delivered without looking at the recipe book in Part 2.	9.01	1.64	10	10	9	10	−2.37	6.59	47.2729^*^	
Number of perseverations when making the ice creams in Part 2	0.83	1.30	0	6	0	1	1.89	3.44	47.2739^*^	
Learning potential in terms of flexibility when making ice cream #4 in Part 2 (which was ice cream #1 in Part 1)	93.20	50.90	97	147	54	147	−0.55	−1.00	18.7337^*^	
Learning potential in terms of flexibility when making ice cream #1 in Part 2 (which is different from ice cream #1 in Part 1)	79.23	48.57	98	125	31	125	−0.45	−1.40	30.7827^*^	

The sample size is 398 and the minimum of each variable is 0.

* All variables show “NOT normality” with a *p* < 0.001.

### 3.5. Validity and reliability of the scales

Validity is the result of a process of gathering empirical evidence based on theoretical assumptions that, in sum, allow us to make an evaluative judgment that affirms the relevance and sufficiency of the interpretations based on the results of a test. This judgment depends not only on the items of the test, but also on the sample on which the test is carried out, and on the context of application.

Construct validity is the unifying concept that integrates content and criterion validity considerations into a common framework for testing hypotheses about theoretically relevant relationships (Messick, 1980). The ultimate goal of validation is explanation and understanding, and therefore, this leads us to consider that all validation is construct validation (Cronbach, 1951). The most widely used methodological procedures for obtaining data on the validity of psychological constructs have been factor analysis and the multitrait-multimethod matrix. Both systems are respective indicators of the so-called “factorial validity” and “convergent-discriminant validity.”

**Figure 5:**
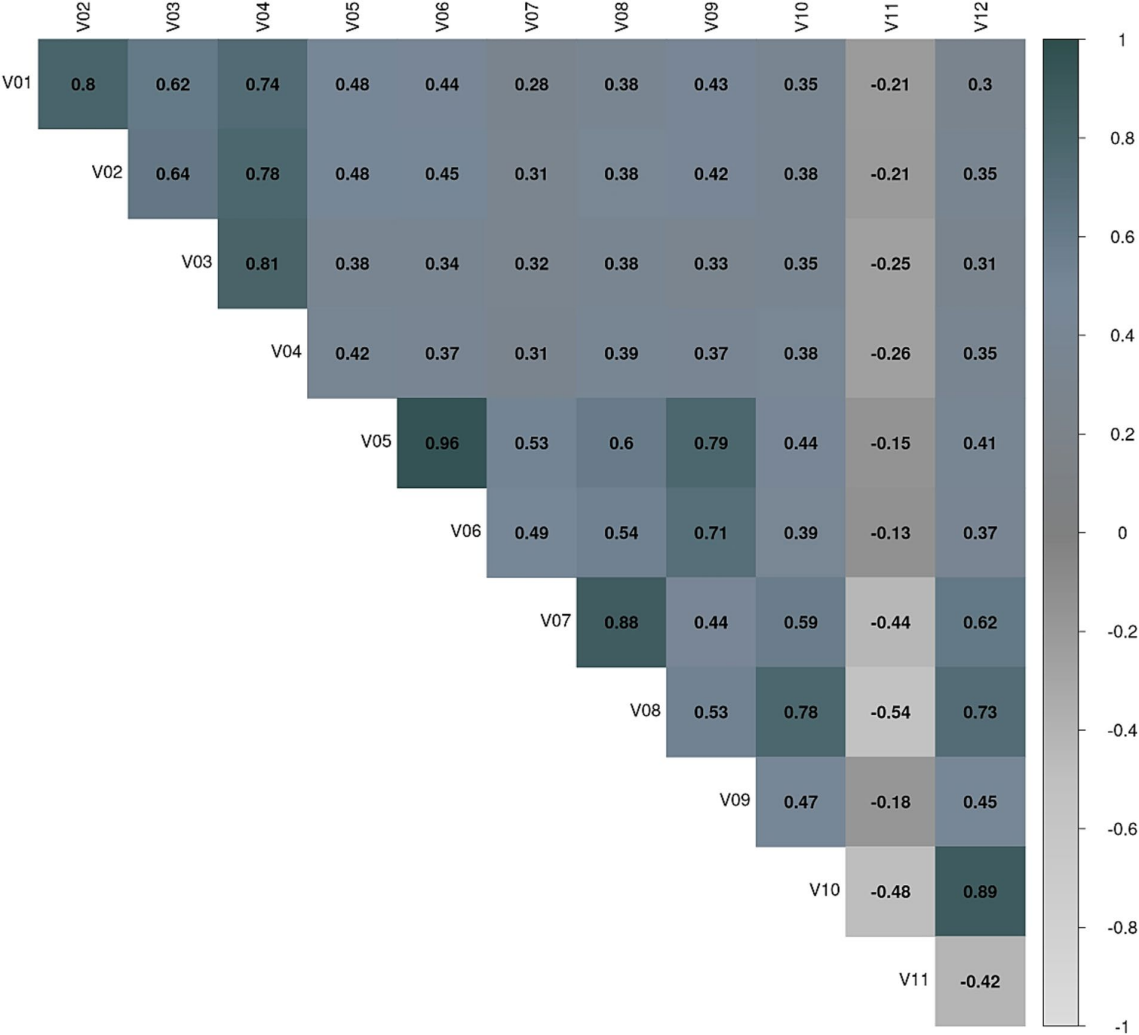
Ice Cream VR test. Variable correlation matrix. V01: Number of shifts correctly assigned in Part 1. V02: Number of shifts correctly assigned in Part 2. V03: Learning potential to identify whether the customer wears a neoprene suit or not, (measured at Round 13). V04: Learning potential when it comes to assign the right order to the customers. V05: s1.h.score.n. V06: Number of correct #1 ice creams delivered without looking at the recipe book in Part 1 rounds. V07: Number of correct #1 ice creams delivered without looking at the recipe book in Part 2. V08: Number of total correct ice creams delivered correctly without looking at the recipe book on Part 2 rounds. V09: Learning potential in relation to making ice cream #1 correctly. V10: Learning potential in terms of flexibility when making ice cream #4 in Part 2 (which was ice cream #1 in Part 1). V11: Number of perseverations when making the ice creams in Part 2. V12: Learning potential in terms of flexibility when making ice cream #1 in Part 2 (which is different from ice cream #1 in Part 1).

For this study, convergent-discriminant validity will not be addressed because all the variables are part of one of the constructs and there is also a relationship between them. The basic underlying assumptions of factor analysis are more conceptual than statistical. From this point of view, the assumptions of normality and homoscedasticity can be ignored, being aware that their non-compliance produces a decrease in the observed correlations. In reality, normality is only necessary when a statistical test is applied to the significance of the factors; however, such tests are rarely used. In fact, some degree of multicollinearity is desirable. If visual inspection reveals that there is not a substantial number of correlations greater than 0.30 then the factor analysis is probably inappropriate (Cronbach, 1988). The following Figure 5 shows that this is not the case.

The presence of multicollinearity can be identified by evaluating the determinant of the correlation matrix of the variables entered into the study: A low determinant, i.e., close to 0, indicates high multicollinearity between the variables. Barlett’s test of sphericity is obtained by a transformation of the determinant of the correlation matrix and compares, under the hypothesis of multivariate normality, whether the correlation matrix of the p variables observed is the identity. If a correlation matrix is the identity, it means that the intercorrelations between the variables are zero. If the null hypothesis is confirmed, the variables are not intercorrelated. Conversely, if the test statistic shows large values (or a determinant close to zero) the null hypothesis is rejected with some degree of significance. If the null hypothesis is accepted, the variables are not intercorrelated and the application of a factor analysis should be reconsidered. These results (Barlett Statistic = 1147.46, df = 66, *p* < 0.000) implied the existence of correlated variables and, therefore, indicate a factor analysis can be applied.

### 3.6. Factor analysis

As a next step to confirm the feasibility of performing a factor analysis, a sample adequacy analysis was performed. Sample adequacy measures whether the variables share common factors. In short, if there are a large number of non-zero partial correlation coefficients, it is interpreted that the hypotheses of the factor model are not compatible with the data (Shrestha, 2021). One way to quantify this fact is with Kaiser–Meyer–Olkin’s KMO Sample Mean of Adequacy. A KMO value of less than 0.5 indicates that it is not acceptable to carry out a factor analysis with the data provided. In this case, as shown in Table 14, all values obtained were higher than 0.75 (KMO = 0.82).

**Table 14:** Sample adequacy means.

Variable	KMO
Number of shifts correctly assigned in Part 1	0.89
Number of shifts correctly assigned in Part 2	0.88
Learning potential to identify whether the customer wears a neoprene suit	0.85
Learning potential when it comes to assign the right order to the customers	0.83
Number of total correct ice creams delivered correctly without looking at the recipe book on Part 1 rounds	0.75
Number of correct #1 ice creams delivered without looking at the recipe book in Part 1 rounds.	0.77
Number of correct #1 ice creams delivered without looking at the recipe book in Part 2.	0.76
Number of total correct ice creams delivered correctly without looking at the recipe book on Part 2 rounds	0.78
Learning potential in relation to making ice cream #1 correctly	0.90
Learning potential in terms of flexibility when making ice cream #4 in Part 2 (which was ice cream #1 in Part 1)	0.75
Number of perseverations when making the ice creams in Part 2	0.91
Learning potential in terms of flexibility when making ice cream #1 in Part 2 (which is different from ice cream #1 in Part 1)	0.80

Kaiser–Meyer–Olkin.

Therefore, it is acceptable to perform a factor analysis. The results of the factor analysis were as shown below in Table 15.

**Table 15:** Factor analysis results.

Variable	Planning	Learning	Flexibility
Number of shifts correctly assigned in Part 1	**0.787**	0.108	−0.051
Number of shifts correctly assigned in Part 2	**0.823**	0.094	−0.051
Learning potential to identify whether the customer wears a neoprene suit	**0.846**	−0.066	0.022
Learning potential when it comes to assign the right order to the customers	**0.993**	−0.072	−0.029
Number of total correct ice creams delivered correctly without looking at the recipe book on Part 1 rounds	0.004	**0.958**	0.073
Number of correct #1 ice creams delivered without looking at the recipe book in Part 1 rounds.	−0.02	**0.955**	0.026
Number of correct #1 ice creams delivered without looking at the recipe book in Part 2.	−0.112	0.112	**0.871**
Number of total correct ice creams delivered correctly without looking at the recipe book on Part 2 rounds	−0.059	0.104	**0.968**
Learning potential in relation to making ice cream #1 correctly	0.048	**0.689**	0.137
Learning potential in terms of flexibility when making ice cream #4 in Part 2 (which was ice cream #1 in Part 1)	0.082	−0.016	**0.761**
Number of perseverations when making the ice creams in Part 2	−0.092	0.24	**−0.638**
Learning potential in terms of flexibility when making ice cream #1 in Part 2 (which is different from ice cream #1 in Part 1)	0.053	−0.006	**0.724**

Bold values show the highest weight for each variable.

The factor loadings matrix plays an important role in interpreting the meaning of the factors. When the factors are orthogonal they quantify the degree and type of the relationship between the factors and the original variables. In practice, factor extraction methods may not provide adequate factor loading matrices for interpretation. In order to tackle this problem, there are factor rotation procedures which, starting from the initial solution, search for factors whose factor loadings matrix makes them more easily interpretable. Of the three procedures used: orthogonal, varimax and promax, it is the promax rotation procedure that has allowed a better interpretation of the loading of the variables in the factors. The promax procedure alters the results of an orthogonal rotation to create a solution with factor loadings as close as possible to the ideal structure. The ideal structure is obtained by raising to a power (between 2 and 4) the factorial loadings obtained in an orthogonal rotation. The higher the power, the more oblique the solution obtained.

The Factorial Analysis carried out explains 72.4% of the variance. Separately, the percentage of variance that has not been explained by the three factors (‘planning’, ‘learning’, ‘flexibility’) is shown in Supplementary Table 3.

### 3.7. Test reliability and internal consistency

The Ice Cream test presents certain special characteristics that, in some respects, bring it closer to an “adaptive” type of test, since the time of presentation between stimuli, the appearance of distractors, their frequency, etc. depend on the sequence of responses given by the person. In many respects it could be said that each subject may actually be responding to a “different” test. This, which considerably improves the ecological validity of the test and its real efficacy, makes it difficult, however, to estimate the reliability of all the measures scaled, at least in what is traditionally understood as the reliability coefficient of a test. This is the reason why it is only possible to estimate the classical reliability of scales. Nevertheless, if these are reliable, in turn, they also guarantee the reliability of the rest of the aspects scaled. It should also be clarified that aspects such as standard deviations, reaction times, etc., which can be very useful for the diagnosis and classification of adults, do not support, strictly speaking, the concept of reliability coefficient.

To determine the absence of errors in the measurement of a test, or the precision of its measurement, that is, its reliability, Cronbach’s alpha will be used. This is the degree to which all test items co-vary with each other. Cronbach’s alpha is not a usual statistic, so it is not accompanied by any *p*-value that allows us to reject the hypothesis of reliability in the scale, but the alpha is accompanied by its corresponding 95% confidence interval. However, the closer it is to its maximum value, 1, the greater the reliability of the scale. Furthermore, in certain contexts and by tacit agreement, it is considered that alpha values greater than 0.7 or 0.8 (depending on the source) are sufficient to guarantee the reliability of the scale. An alternative method for reliability estimation is McDonald’s omega which works with factor loadings that are the weighted sum of the standardized variables, a transformation that makes the calculations more stable (Ventura Leon and Caycho-Rodríguez, 2017) and assumes that the variance between items can be different. The difficulty index and discrimination index have also been calculated. These indices become indicators of the quality of a test to the extent that they are within acceptable ranges. The difficulty index measures the difficulty of an item, and the discrimination index is the power of an item to distinguish between subjects who perform the task well and those who do not. Note that it is common to find in the literature the “difficulty index” or “degree of difficulty” as the ratio between the number of correct answers and the maximum possible score. According to this definition, the higher the index, the higher the number of correct answers and therefore the easier the question, which is the opposite of difficulty. From a purely semantic point of view, it is more accurate to call the ratio between the number of correct answers and the total number of examinees an ease index, as explained by García-Cueto and Fidalgo (2005). Data for Test Reliability and Internal Consistency are provided in Supplementary Table 4.

## 4. Discussion

The present study has presented the first data that were obtained for Nesplora Ice Cream as a new ecological, virtual reality-based test for the obtention of a comprehensive profile of executive functions. More specifically, the data presented here are the first set of normative data collected for children between 8 and 16 years old, thus becoming, to our knowledge, in the first tool of its kind (a VR-based neuropsychological test for executive functions) in providing normative data of this magnitude for this age range (i.e., children and adolescents).

Among the extensive number of variables potentially produced by the test, the current normative study has tried to show the main core variables measured by the test. As a consequence, the statistical procedures leading to a confirmatory factor analysis have reduced the existing measures into 12 main core measures that divide precisely into 3 factors, namely Planning (4 measures), Learning (3 measures) and Cognitive Flexibility (5 measures). These three factors explain more than 72% of the variance. Cluster analyses carried out have also shown that the recommendation for the establishment of two differentiated age groups for Planning and Cognitive Flexibility (Group 1: 8 to 11 years-old; group 2: 12 to 16 years-old), and for Learning (Group 1: 8 to 9 years-old; group 2: 10 to 16 years-old) give clues on the milestones for development of executive functions in these stages of development.

Additionally, cluster analyses by gender have shown no statistically significant differences between boys and girls, which makes it unnecessary to establish separate normative groups by gender. Moreover, reliability and internal consistency data are presented, and specific ceiling and floor effects detected per each scale x age-group combination have been reported.

Despite the limitations of the current normative study (focused on population from Spain, and thus requiring as a priority for immediate future research a cross-cultural validation that allows its administration and clinical use in different international settings), the Nesplora Ice Cream VR test implies a clear hamper of ecological validity as described by Marcotte et al. (2010). As pointed out by Diaz-Orueta et al. (2022), VR-based tests like this (1) overcome the limitations of traditional sterile, distractor-free testing environments that do not capture real-life environmental demands, allowing a more accurate prediction of an individual’s level of function in real-life settings; (2) allow the monitoring of testee’s behavior in a more continuous way, increasing the sample of behavior usually captured by traditional standardized neuropsychological tests; and (3) provide more clarity to the nature of specific cognitive constructs measured, which *per se* is an innovation in the area of executive functions tests, by properly delineating the boundaries between planning, learning and cognitive flexibility measures. Separately, since the focus on the 8 to 16 years old group cannot provide a full picture on the trajectories of EF development, additional studies would be required with a more detailed focus on the use of the test to uncover the developmental trajectories of EF across the lifespan, which would require a comparison between different cohorts that falls beyond the scope of the current study. Moreover, the statistical procedures followed in the study (i.e., cluster analysis and confirmatory factor analysis) mainly focus on a construct validity approach, and further convergent validity studies -as well as studies with specific clinical populations -would be desirable to prove further the added value of this test versus traditional EF measures.

In relation to previous attempts to improve ecological validity, the most reliable example of an executive function test aiming for accurately predict behavior based on its results is the Behavioral Assessment of the Dysexecutive Syndrome (BADS; Wilson et al., 1996) for evaluation of executive functions, and the Naturalistic Action Test (NAT; Giovannetti et al., 2002) for the assessment of level of independent functioning. However, developments in the area of VR, subject to adequate quality, allow both clinicians and researchers to administer ecologically relevant stimuli placed in a meaningful and familiar context and, as a result, they can measure responses and behaviors in a more comprehensive way (provided visual and physical characteristics of items, avatars and characters are of high quality and realistic). Additionally, as previously pointed out by Diaz-Orueta et al. (2022), VR technology allows tester-control over stimuli, distractors and other variables, and any or all of these factors can be adjusted depending on the response features of the individual undergoing assessment – thereby allowing more personalized assessment.

In summary, this study, despite the constraints and the need for cross-cultural validation with additional, international community-based and clinical samples, constitutes, to our best knowledge, the first Virtual Reality based neuropsychological test that provides normative data for the age group of 8 to 16 years old that measures and distinguishes in a meaningful, ecological way between planning, learning and cognitive flexibility processes. Future additional research is needed to ensure that these measures allow reliable and accurate predictions that extend the application of these types of tools to early detection of executive syndromes and subsequent appropriate treatment planning and accurate prediction of behavioral outcomes in different clinical settings with different conditions affecting executive functioning.

## Data availability statement

The raw data supporting the conclusions of this article will be made available by the authors, without undue reservation.

## Ethics statement

The studies involving humans were approved by Ethics Committee related to Research with Human Beings from the University of the Basque Country (UPV-EHU), Spain. The studies were conducted in accordance with the local legislation and institutional requirements. Written informed consent for participation in this study was provided by the participants’ legal guardians/next of kin.

## Author contributions

MF, GC, and UD-O conceptualized the study and wrote the first draft. MF conducted the first literature review, and UD-O completed it. UD-O described the measure used and MS-C completed it. MS-C worked on the recruitment, data collection and ethical issues of the study. FR-O produced all the statistical analysis, with collaboration from MF, GC, and UD-O. MF and UD-O wrote the discussion and refined the final draft. All authors contributed to the article and approved the submitted version.
