# Supplementary material for: Ice Cream: new virtual reality tool for the assessment of executive functions in children and adolescents: a normative study
Source: Front Psychol. 2023 Sep 22;14:1196964. doi: 10.3389/fpsyg.2023.1196964 (PMC10556863; doi:10.3389/fpsyg.2023.1196964)
Supplement: Supplementary file 1 [file Table_1.docx]

**Supplementary Table 1. General abbreviations in the Ice Cream VR test report**

| Abbreviation (prefix-suffix) | Description |
| --- | --- |
| t. | Totals |
| s1. | Part or set 1 |
| s2. | Part or set 2 |
| r00. | Round or turn, and number. Ranges from 0 to 14  [0 is Training]. |
| .a00. | Avatar number. Ranks from 0 to 3. |
| .h. | Ice cream |
| ...turn. \| tu | Shift |
| .n | The variable stores a value that it returns. |

**Supplementary Table 2. Clinical Report Main Variables**

| Variable | | Description | |
| --- | --- | --- | --- |
| s1_tu_total_correct_n | | Number of shifts correctly assigned in Part 1 | |
| s2_tu_total_correct_n | | Number of shifts correctly assigned in Part 2 | |
| R01:14_axx:axx_n_performance | | Neoprene avatar criterion learning potential score Rounds 1 to 14 | |
| R01:14_axx:axx_t_performance | | Learning potential score avatar ticket criterion in Rounds 1 to 14 | |
| s1_h_score_n | | Number of total correct ice creams delivered correctly without looking at the recipe book on Part 1 rounds | |
| s2_h_score_n | | Number of total correct ice creams delivered correctly without looking at the recipe book on Part 2 rounds | |
| R01:07_axx:axx_h1_performance | | Learning potential score for ice cream #1 in Part 1 (Rounds 1 to 7) | |
| R08:14_axx:axx_h4_performance | | Learning Potential Score for Ice Cream #4 at Rounds 8 to 14 | |
| R08:14_axx:axx_h1_performance | | Learning potential score for ice cream #1 at Rounds 8 to 14 | |
| s1_h1_score_n | | Number of correct #1 ice creams delivered without looking at the recipe book in Part 1 rounds | |
| s2_h1_score_n | | Number of correct #1 ice creams delivered without looking at the recipe book in Part 2 | |
| s2_h_persepress_n | | Indicates that a #3 is made when a #1 should be made in Part 2 (same ice cream ingredients but different ice cream number in Part 2, indicative of perseveration). | |
| rx.tu.total.correct.n | | Indicates the number of times the order of clients has been correctly set based on the rules set by the instructions | |
| rx.ax.turn.info.is.correct.n | | Indicates if the given position in the queue is the correct one for that avatar in that turn | |
| rx.h.rawscore.n | | Number of ice cream delivered correctly, regardless of whether or not the reference is consulted in turn. | |
| rx.ax.h.info.is.correct.n | | Indicates whether an avatar has been given the correct ice cream in their round. | |
|  |  | |  |

**Supplementary Table 3. Unexplained Variance.**

| Variable | Uniqueness |
| --- | --- |
| Number of shifts correctly assigned in Part 1 | 0.33 |
| Number of shifts correctly assigned in Part 2 | 0.28 |
| Learning potential to identify whether the customer wears a neoprene suit | 0.32 |
| Learning potential when it comes to assign the right order to the customers | 0.10 |
| Number of total correct ice creams delivered correctly without looking at the recipe book on Part 1 rounds | 0.00 |
| Number of correct #1 ice creams delivered without looking at the recipe book in Part 1 rounds. | 0.08 |
| Number of correct #1 ice creams delivered without looking at the recipe book in Part 2. | 0.23 |
| Number of total correct ice creams delivered correctly without looking at the recipe book on Part 2 rounds | 0.01 |
| Learning potential in relation to making ice cream #1 correctly | 0.37 |
| Learning potential in terms of flexibility when making ice cream #4 in Part 2 (which was ice cream #1 in Part 1) | 0.37 |
| Number of perseverations when making the ice creams in Part 2 | 0.64 |
| Learning potential in terms of flexibility when making ice cream #1 in Part 2 (which is different from ice cream #1 in Part 1) | 0.44 |

**Supplementary Table 4. Test Reliability and Internal Consistency**

|  | easiness | discrimination | alpha2.5 | alpha | alpha97.5 | omega |
| --- | --- | --- | --- | --- | --- | --- |
| r01.tu.total.correct.n | 0.49 | 0.71 | 0.91 | 0.92 | 0.92 | 0.94 |
| r02.tu.total.correct.n | 0.67 | 0.63 | 0.91 | 0.92 | 0.92 | 0.94 |
| r03.tu.total.correct.n | 0.57 | 0.89 | 0.91 | 0.92 | 0.92 | 0.94 |
| r04.tu.total.correct.n | 0.83 | 0.42 | 0.91 | 0.92 | 0.92 | 0.94 |
| r05.tu.total.correct.n | 0.78 | 0.56 | 0.91 | 0.92 | 0.92 | 0.94 |
| r06.tu.total.correct.n | 0.60 | 0.90 | 0.91 | 0.92 | 0.92 | 0.94 |
| r07.tu.total.correct.n | 0.74 | 0.72 | 0.91 | 0.92 | 0.92 | 0.94 |
| r08.tu.total.correct.n | 0.61 | 0.93 | 0.91 | 0.92 | 0.92 | 0.94 |
| r09.tu.total.correct.n | 0.70 | 0.75 | 0.91 | 0.92 | 0.92 | 0.94 |
| r10.tu.total.correct.n | 0.70 | 0.66 | 0.91 | 0.92 | 0.92 | 0.94 |
| r11.tu.total.correct.n | 0.67 | 0.61 | 0.91 | 0.92 | 0.92 | 0.94 |
| r12.tu.total.correct.n | 0.67 | 0.80 | 0.91 | 0.92 | 0.92 | 0.94 |
| r13.tu.total.correct.n | 0.69 | 0.81 | 0.91 | 0.92 | 0.92 | 0.94 |
| r14.tu.total.correct.n | 0.60 | 0.90 | 0.91 | 0.92 | 0.92 | 0.94 |
| r01.a00.turn.info.is.correct.n | 0.76 | 0.28 | 0.96 | 0.97 | 0.97 | 0.97 |
| r01.a01.turn.info.is.correct.n | 0.55 | 0.59 | 0.96 | 0.97 | 0.97 | 0.97 |
| r01.a02.turn.info.is.correct.n | 0.63 | 0.67 | 0.96 | 0.97 | 0.97 | 0.97 |
| r01.a03.turn.info.is.correct.n | 0.87 | 0.27 | 0.96 | 0.97 | 0.97 | 0.97 |
| r02.a00.turn.info.is.correct.n | 0.92 | 0.22 | 0.96 | 0.97 | 0.97 | 0.97 |
| r02.a01.turn.info.is.correct.n | 0.71 | 0.50 | 0.96 | 0.97 | 0.97 | 0.97 |
| r02.a02.turn.info.is.correct.n | 0.69 | 0.55 | 0.96 | 0.97 | 0.97 | 0.97 |
| r02.a03.turn.info.is.correct.n | 0.88 | 0.30 | 0.96 | 0.97 | 0.97 | 0.97 |
| r03.a00.turn.info.is.correct.n | 0.63 | 0.76 | 0.96 | 0.97 | 0.97 | 0.97 |
| r03.a01.turn.info.is.correct.n | 0.67 | 0.66 | 0.96 | 0.97 | 0.97 | 0.97 |
| r03.a02.turn.info.is.correct.n | 0.79 | 0.55 | 0.96 | 0.97 | 0.97 | 0.97 |
| r03.a03.turn.info.is.correct.n | 0.66 | 0.71 | 0.96 | 0.97 | 0.97 | 0.97 |
| r04.a00.turn.info.is.correct.n | 0.88 | 0.32 | 0.96 | 0.97 | 0.97 | 0.97 |
| r04.a01.turn.info.is.correct.n | 0.86 | 0.35 | 0.96 | 0.97 | 0.97 | 0.97 |
| r04.a02.turn.info.is.correct.n | 0.88 | 0.28 | 0.96 | 0.97 | 0.97 | 0.97 |
| r04.a03.turn.info.is.correct.n | 0.94 | 0.16 | 0.96 | 0.97 | 0.97 | 0.97 |
| r05.a00.turn.info.is.correct.n | 0.85 | 0.41 | 0.96 | 0.97 | 0.97 | 0.97 |
| r05.a01.turn.info.is.correct.n | 0.85 | 0.38 | 0.96 | 0.97 | 0.97 | 0.97 |
| r05.a02.turn.info.is.correct.n | 0.84 | 0.43 | 0.96 | 0.97 | 0.97 | 0.97 |
| r05.a03.turn.info.is.correct.n | 0.82 | 0.49 | 0.96 | 0.97 | 0.97 | 0.97 |
| r06.a00.turn.info.is.correct.n | 0.84 | 0.45 | 0.96 | 0.97 | 0.97 | 0.97 |
| r06.a01.turn.info.is.correct.n | 0.71 | 0.79 | 0.96 | 0.97 | 0.97 | 0.97 |
| r06.a02.turn.info.is.correct.n | 0.66 | 0.74 | 0.96 | 0.97 | 0.97 | 0.97 |
| r06.a03.turn.info.is.correct.n | 0.66 | 0.78 | 0.96 | 0.97 | 0.97 | 0.97 |
| r07.a00.turn.info.is.correct.n | 0.83 | 0.48 | 0.96 | 0.97 | 0.97 | 0.97 |
| r07.a01.turn.info.is.correct.n | 0.84 | 0.45 | 0.96 | 0.97 | 0.97 | 0.97 |
| r07.a02.turn.info.is.correct.n | 0.79 | 0.59 | 0.96 | 0.97 | 0.97 | 0.97 |
| r07.a03.turn.info.is.correct.n | 0.81 | 0.54 | 0.96 | 0.97 | 0.97 | 0.97 |
| r08.a00.turn.info.is.correct.n | 0.68 | 0.84 | 0.96 | 0.97 | 0.97 | 0.97 |
| r08.a01.turn.info.is.correct.n | 0.69 | 0.77 | 0.96 | 0.97 | 0.97 | 0.97 |
| r08.a02.turn.info.is.correct.n | 0.83 | 0.45 | 0.96 | 0.97 | 0.97 | 0.97 |
| r08.a03.turn.info.is.correct.n | 0.66 | 0.78 | 0.96 | 0.97 | 0.97 | 0.97 |
| r09.a00.turn.info.is.correct.n | 0.89 | 0.23 | 0.96 | 0.97 | 0.97 | 0.97 |
| r09.a01.turn.info.is.correct.n | 0.77 | 0.54 | 0.96 | 0.97 | 0.97 | 0.97 |
| r09.a02.turn.info.is.correct.n | 0.87 | 0.35 | 0.96 | 0.97 | 0.97 | 0.97 |
| r09.a03.turn.info.is.correct.n | 0.73 | 0.74 | 0.96 | 0.97 | 0.97 | 0.97 |
| r10.a00.turn.info.is.correct.n | 0.92 | 0.17 | 0.96 | 0.97 | 0.97 | 0.97 |
| r10.a01.turn.info.is.correct.n | 0.74 | 0.58 | 0.96 | 0.97 | 0.97 | 0.97 |
| r10.a02.turn.info.is.correct.n | 0.84 | 0.41 | 0.96 | 0.97 | 0.97 | 0.97 |
| r10.a03.turn.info.is.correct.n | 0.76 | 0.51 | 0.96 | 0.97 | 0.97 | 0.97 |
| r11.a00.turn.info.is.correct.n | 0.78 | 0.39 | 0.96 | 0.97 | 0.97 | 0.97 |
| r11.a01.turn.info.is.correct.n | 0.71 | 0.53 | 0.96 | 0.97 | 0.97 | 0.97 |
| r11.a02.turn.info.is.correct.n | 0.79 | 0.47 | 0.96 | 0.97 | 0.97 | 0.97 |
| r11.a03.turn.info.is.correct.n | 0.87 | 0.32 | 0.96 | 0.97 | 0.97 | 0.97 |
| r12.a00.turn.info.is.correct.n | 0.71 | 0.74 | 0.96 | 0.97 | 0.97 | 0.97 |
| r12.a01.turn.info.is.correct.n | 0.77 | 0.61 | 0.96 | 0.97 | 0.97 | 0.97 |
| r12.a02.turn.info.is.correct.n | 0.75 | 0.62 | 0.96 | 0.97 | 0.97 | 0.97 |
| r12.a03.turn.info.is.correct.n | 0.90 | 0.20 | 0.96 | 0.97 | 0.97 | 0.97 |
| r13.a00.turn.info.is.correct.n | 0.86 | 0.37 | 0.96 | 0.97 | 0.97 | 0.97 |
| r13.a01.turn.info.is.correct.n | 0.79 | 0.60 | 0.96 | 0.97 | 0.97 | 0.97 |
| r13.a02.turn.info.is.correct.n | 0.73 | 0.71 | 0.96 | 0.97 | 0.97 | 0.97 |
| r13.a03.turn.info.is.correct.n | 0.70 | 0.79 | 0.96 | 0.97 | 0.97 | 0.97 |
| r14.a00.turn.info.is.correct.n | 0.68 | 0.80 | 0.96 | 0.97 | 0.97 | 0.97 |
| r14.a01.turn.info.is.correct.n | 0.68 | 0.79 | 0.96 | 0.97 | 0.97 | 0.97 |
| r14.a02.turn.info.is.correct.n | 0.67 | 0.75 | 0.96 | 0.97 | 0.97 | 0.97 |
| r14.a03.turn.info.is.correct.n | 0.90 | 0.22 | 0.96 | 0.97 | 0.97 | 0.97 |
| r01.h.rawscore.n | 0.69 | 0.44 | 0.73 | 0.75 | 0.75 | 0.80 |
| r02.h.rawscore.n | 0.83 | 0.32 | 0.73 | 0.75 | 0.75 | 0.80 |
| r03.h.rawscore.n | 0.80 | 0.38 | 0.73 | 0.75 | 0.75 | 0.80 |
| r04.h.rawscore.n | 0.91 | 0.20 | 0.73 | 0.75 | 0.75 | 0.80 |
| r05.h.rawscore.n | 0.90 | 0.25 | 0.73 | 0.75 | 0.75 | 0.80 |
| r06.h.rawscore.n | 0.94 | 0.16 | 0.73 | 0.75 | 0.75 | 0.80 |
| r07.h.rawscore.n | 0.93 | 0.13 | 0.73 | 0.75 | 0.75 | 0.80 |
| r08.h.rawscore.n | 0.59 | 0.50 | 0.73 | 0.75 | 0.75 | 0.80 |
| r09.h.rawscore.n | 0.49 | 0.63 | 0.73 | 0.75 | 0.75 | 0.80 |
| r10.h.rawscore.n | 3.53 | 1.04 | 0.73 | 0.75 | 0.75 | 0.80 |
| r11.h.rawscore.n | 0.71 | 0.58 | 0.73 | 0.75 | 0.75 | 0.80 |
| r12.h.rawscore.n | 0.69 | 0.60 | 0.73 | 0.75 | 0.75 | 0.80 |
| r13.h.rawscore.n | 0.76 | 0.55 | 0.73 | 0.75 | 0.75 | 0.80 |
| r14.h.rawscore.n | 0.78 | 0.50 | 0.73 | 0.75 | 0.75 | 0.80 |
| r01.a00.h.info.is.correct.n | 0.90 | 0.18 | 0.83 | 0.85 | 0.85 | 0.87 |
| r01.a01.h.info.is.correct.n | 0.90 | 0.15 | 0.83 | 0.85 | 0.85 | 0.87 |
| r01.a02.h.info.is.correct.n | 0.88 | 0.23 | 0.83 | 0.85 | 0.85 | 0.87 |
| r01.a03.h.info.is.correct.n | 0.89 | 0.19 | 0.83 | 0.85 | 0.85 | 0.87 |
| r02.a00.h.info.is.correct.n | 0.91 | 0.14 | 0.83 | 0.85 | 0.85 | 0.87 |
| r02.a01.h.info.is.correct.n | 0.95 | 0.11 | 0.83 | 0.85 | 0.85 | 0.87 |
| r02.a02.h.info.is.correct.n | 0.94 | 0.15 | 0.83 | 0.85 | 0.85 | 0.87 |
| r02.a03.h.info.is.correct.n | 0.94 | 0.13 | 0.83 | 0.85 | 0.85 | 0.87 |
| r03.a00.h.info.is.correct.n | 0.95 | 0.10 | 0.83 | 0.85 | 0.85 | 0.87 |
| r03.a01.h.info.is.correct.n | 0.92 | 0.18 | 0.83 | 0.85 | 0.85 | 0.87 |
| r03.a02.h.info.is.correct.n | 0.95 | 0.10 | 0.83 | 0.85 | 0.85 | 0.87 |
| r03.a03.h.info.is.correct.n | 0.90 | 0.23 | 0.83 | 0.85 | 0.85 | 0.87 |
| r04.a00.h.info.is.correct.n | 0.95 | 0.12 | 0.83 | 0.85 | 0.85 | 0.87 |
| r04.a01.h.info.is.correct.n | 0.98 | 0.04 | 0.83 | 0.85 | 0.85 | 0.87 |
| r04.a02.h.info.is.correct.n | 0.97 | 0.07 | 0.83 | 0.85 | 0.85 | 0.87 |
| r04.a03.h.info.is.correct.n | 0.97 | 0.07 | 0.83 | 0.85 | 0.85 | 0.87 |
| r05.a00.h.info.is.correct.n | 0.98 | 0.06 | 0.83 | 0.85 | 0.85 | 0.87 |
| r05.a01.h.info.is.correct.n | 0.98 | 0.04 | 0.83 | 0.85 | 0.85 | 0.87 |
| r05.a02.h.info.is.correct.n | 0.98 | 0.05 | 0.83 | 0.85 | 0.85 | 0.87 |
| r05.a03.h.info.is.correct.n | 0.93 | 0.16 | 0.83 | 0.85 | 0.85 | 0.87 |
| r06.a00.h.info.is.correct.n | 0.96 | 0.10 | 0.83 | 0.85 | 0.85 | 0.87 |
| r06.a01.h.info.is.correct.n | 0.99 | 0.04 | 0.83 | 0.85 | 0.85 | 0.87 |
| r06.a02.h.info.is.correct.n | 0.98 | 0.05 | 0.83 | 0.85 | 0.85 | 0.87 |
| r06.a03.h.info.is.correct.n | 0.99 | 0.03 | 0.83 | 0.85 | 0.85 | 0.87 |
| r07.a00.h.info.is.correct.n | 0.97 | 0.05 | 0.83 | 0.85 | 0.85 | 0.87 |
| r07.a01.h.info.is.correct.n | 0.99 | 0.02 | 0.83 | 0.85 | 0.85 | 0.87 |
| r07.a02.h.info.is.correct.n | 0.98 | 0.05 | 0.83 | 0.85 | 0.85 | 0.87 |
| r07.a03.h.info.is.correct.n | 0.98 | 0.05 | 0.83 | 0.85 | 0.85 | 0.87 |
| r08.a00.h.info.is.correct.n | 0.81 | 0.30 | 0.83 | 0.85 | 0.85 | 0.87 |
| r08.a01.h.info.is.correct.n | 0.81 | 0.21 | 0.83 | 0.85 | 0.85 | 0.87 |
| r08.a02.h.info.is.correct.n | 0.90 | 0.21 | 0.83 | 0.85 | 0.85 | 0.87 |
| r08.a03.h.info.is.correct.n | 0.86 | 0.22 | 0.83 | 0.85 | 0.85 | 0.87 |
| r09.a00.h.info.is.correct.n | 0.69 | 0.40 | 0.83 | 0.85 | 0.85 | 0.87 |
| r09.a01.h.info.is.correct.n | 0.90 | 0.22 | 0.83 | 0.85 | 0.85 | 0.87 |
| r09.a02.h.info.is.correct.n | 0.85 | 0.30 | 0.83 | 0.85 | 0.85 | 0.87 |
| r09.a03.h.info.is.correct.n | 0.76 | 0.41 | 0.83 | 0.85 | 0.85 | 0.87 |
| r10.a00.h.info.is.correct.n | 0.92 | 0.17 | 0.83 | 0.85 | 0.85 | 0.87 |
| r10.a01.h.info.is.correct.n | 0.82 | 0.35 | 0.83 | 0.85 | 0.85 | 0.87 |
| r10.a02.h.info.is.correct.n | 0.84 | 0.34 | 0.83 | 0.85 | 0.85 | 0.87 |
| r10.a03.h.info.is.correct.n | 0.96 | 0.11 | 0.83 | 0.85 | 0.85 | 0.87 |
| r11.a00.h.info.is.correct.n | 0.95 | 0.11 | 0.83 | 0.85 | 0.85 | 0.87 |
| r11.a01.h.info.is.correct.n | 0.92 | 0.17 | 0.83 | 0.85 | 0.85 | 0.87 |
| r11.a02.h.info.is.correct.n | 0.83 | 0.33 | 0.83 | 0.85 | 0.85 | 0.87 |
| r11.a03.h.info.is.correct.n | 0.88 | 0.26 | 0.83 | 0.85 | 0.85 | 0.87 |
| r12.a00.h.info.is.correct.n | 0.82 | 0.38 | 0.83 | 0.85 | 0.85 | 0.87 |
| r12.a01.h.info.is.correct.n | 0.89 | 0.26 | 0.83 | 0.85 | 0.85 | 0.87 |
| r12.a02.h.info.is.correct.n | 0.94 | 0.14 | 0.83 | 0.85 | 0.85 | 0.87 |
| r12.a03.h.info.is.correct.n | 0.88 | 0.28 | 0.83 | 0.85 | 0.85 | 0.87 |
| r13.a00.h.info.is.correct.n | 0.95 | 0.13 | 0.83 | 0.85 | 0.85 | 0.87 |
| r13.a01.h.info.is.correct.n | 0.85 | 0.35 | 0.83 | 0.85 | 0.85 | 0.87 |
| r13.a02.h.info.is.correct.n | 0.92 | 0.16 | 0.83 | 0.85 | 0.85 | 0.87 |
| r13.a03.h.info.is.correct.n | 0.94 | 0.16 | 0.83 | 0.85 | 0.85 | 0.87 |
| r14.a00.h.info.is.correct.n | 0.88 | 0.27 | 0.83 | 0.85 | 0.85 | 0.87 |
| r14.a01.h.info.is.correct.n | 0.94 | 0.15 | 0.83 | 0.85 | 0.85 | 0.87 |
| r14.a02.h.info.is.correct.n | 0.97 | 0.10 | 0.83 | 0.85 | 0.85 | 0.87 |
| r14.a03.h.info.is.correct.n | 0.89 | 0.26 | 0.83 | 0.85 | 0.85 | 0.87 |
